# Lethal course of meconium ileus in preterm twins revealing a novel cystic fibrosis mutation (p.Cys524Tyr)

**DOI:** 10.1186/1471-2431-14-13

**Published:** 2014-01-17

**Authors:** Alexander Puzik, Deborah J Morris-Rosendahl, Klaus-Dieter Rückauer, Claudia Otto, Peter Gessler, Ulrich Saueressig, Roland Hentschel

**Affiliations:** 1 Department of Pediatrics, Albert-Ludwigs-University of Freiburg, Mathildenstrasse 1, D 79106 Freiburg, Germany; 2 Institute of Human Genetics, Albert-Ludwigs-University of Freiburg, Breisacher Strasse 33, D 79106 Freiburg, Germany; 3 National Heart and Lung Institute, Imperial College, London SW3 6LY United Kingdom; 4 Department of Pediatric Surgery, Albert-Ludwigs-University of Freiburg, Hugstetter Strasse 55, D 79106 Freiburg, Germany; 5 Institute of Pathology, Albert-Ludwigs-University of Freiburg, Breisacher Strasse 115a, D 79106 Freiburg, Germany; 6 Department of Pediatrics, Klinikum Konstanz, Luisenstrasse 7, 78464 Konstanz, Germany; 7 Institute of Radiology, Albert-Ludwigs-University of Freiburg, Hugstetter Strasse 55, D 79106 Freiburg, Germany; 8 Department of Radiology, Kreiskrankenhaus Emmendingen, Gartenstraße 44, 79312 Emmendingen Germany

**Keywords:** Preterm infant, Meconium ileus, Cystic fibrosis, Mutation, Disease modifying gene locus, Surgery

## Abstract

**Background:**

In term newborns meconium ileus is frequently associated with cystic fibrosis. Reports on meconium ileus in preterm infants being diagnosed with cystic fibrosis early after birth are very scarce. Associations between genotype and phenotype in cystic fibrosis and its particular comorbidities have been reported.

**Case presentation:**

Two extremely preterm twin infants (26 weeks of gestation) born from a Malaysian mother and a Caucasian father were presented with typical signs of meconium ileus. Despite immediate surgery both displayed a unique and finally lethal course. Mutation analysis revealed a novel, probably pathogenic cystic fibrosis mutation, p.Cys524Tyr. The novel mutation might explain the severity of disease next to typical sequelae of prematurity.

**Conclusion:**

Preterm neonates with meconium ileus have to be evaluated for cystic fibrosis beyond ethnical boundaries, but may take devastating clinical courses despite early treatment. The novel, potentially pathogenic CF mutation p.Cys524Tyr might be associated with severe meconium ileus in neonates. Disease-modifying loci are important targets for intestinal comorbidity of cystic fibrosis.

## Background

Reports on patients with significant prematurity, meconium ileus (MI) and cystic fibrosis (CF) are scarce to the best of our knowledge. Given the fact that ethnicity plays an important role in the occurrence of CF it is of interest to note that MI is generally rare in Malaysia, as is CF in Asia [1]. In general, MI in neonates is thought to account for more than 80% of diagnoses of CF, although only 10-20% of CF patients develop MI [2,3].

Gorter et al. (2010) reported that preterm infants more likely experienced MI without CF and had more complex MI (perforation, atresia, necrosis, volvulus) [2]. The outcome after surgery was favourable, whereas outcome after intestinal perforation was associated with poor survival [4]. Interestingly, survival does not differ between CF patients with and without MI, although MI seems to define more severe disease courses [3].

The role of genetic factors influencing severity and outcome of CF is less well defined. Compound heterozygosity and different modifier loci seem to have a similar impact on disease penetrance and comorbidities as the genotype itself [5-7].

We report on the unique and finally lethal course and complications of extremely preterm twins being diagnosed with a new CF mutation and review and discuss the genetic impact on CF.

## Case presentation

Extremely preterm male twins (26 + 6/7 weeks of gestation, birth weight 980 g and 920 g, monochorionic-diamniotic, Malaysian mother, Caucasian father) were referred to us from an affiliated hospital on *day 9 of life* for persistent MI despite repetitive rectal irrigations.

**Figure 1:**
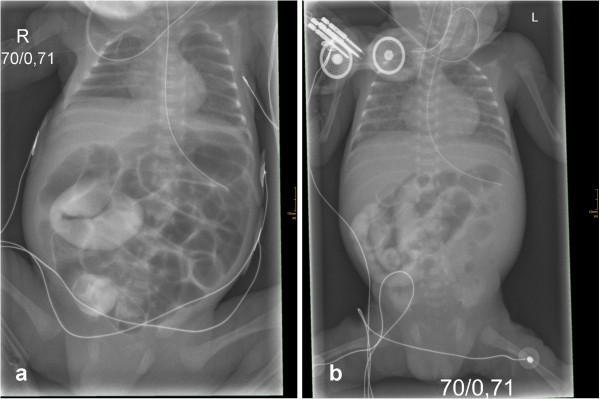
**Initial X-rays of both twins.** Chest and abdominal X-ray with contrast agent upon initial presentation of twin I **(a)** and II **(b)** showing contrast agent stopping at the inflated, distended small intestine next to signs of neonatal respiratory distress syndrome.

**Twin I** displayed good cardiopulmonary adaptation (Apgar score 7/9/9). Upon signs of ileus contrast X-ray of the abdomen (administration per gastric tube) showed immotility of the gut with contrast agent stopping at the inflated, distended small intestine on *day 5* (see Figure 1a).

A laparotomy on *day 10* revealed MI with severe distension of the distal small intestine, microcolon and bowel adhesions. It was essential to form a Bishop-Koop stoma. After starting to pass meconium and tolerating oral feeding initially his haemoglobin level suddenly dropped on *day 28* and ultrasound showed intra-abdominal effusion.

During re-laparotomy multiple hepatic haematoma and bloody ascites were apparent, besides adhesions, multiple coproliths and infarcted and perforated parts of the small intestine. Necrotic parts of the intestine were resected and the Bishop-Koop stoma was converted into two terminal enterostomies. Abdominal wall closure was achieved with a Goretex patch.

In addition, the patient developed respiratory and circulatory failure and consumption coagulopathy. Due to abdominal compartment syndrome the Goretex patch had to be removed forming a laparostoma. He finally displayed refractory metabolic acidosis leading to sudden bradycardia with cardiac arrest.

**Figure 2:**
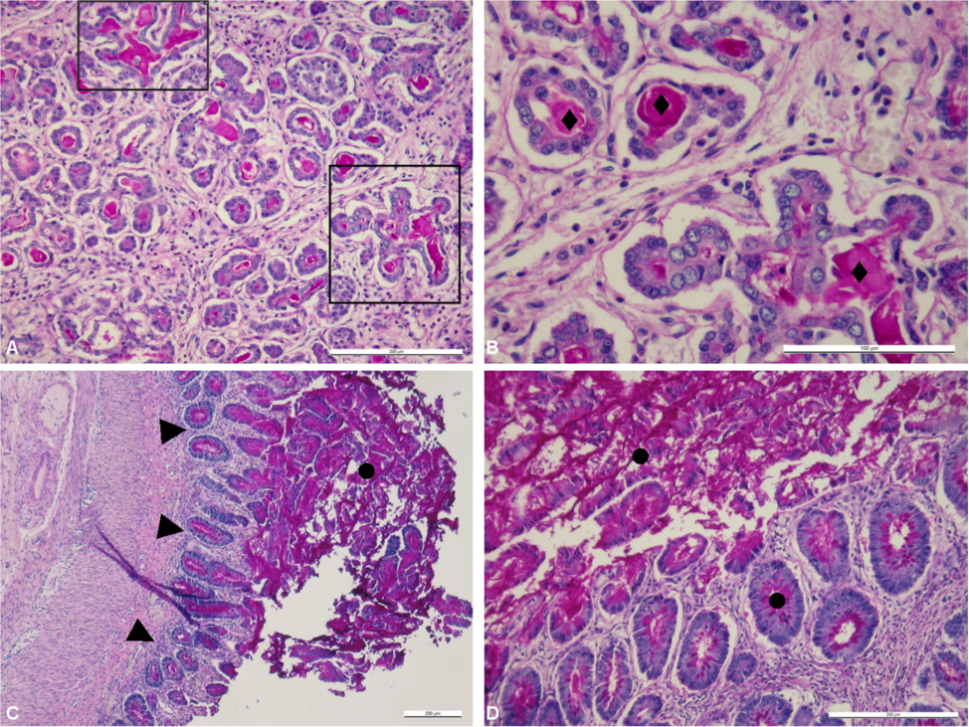
**Autopsy findings of twin I (histology).** Pancreatic tissue shows distinctive PAS-positive intraductal mucous masses (♦) and dilatation of the pancreatic ducts (rectangles). PAS staining, **A** 10×, **B** 20×. Consistent with meconium ileus the large intestine is filled with densely packed stratified mucus (●) while the mucosa is flattened (▲). PAS staining, **C** 4×, **D** 10×.

Autopsy findings of twin I revealed heavily impacted mucus in the small intestine and in crypts of the whole colon. Pancreas histology showed dilated acini and ducts filled up with mucus (see Figure 2a-d).

**Twin II** developed respiratory failure after birth necessitating mechanical ventilation and surfactant administration (Apgar score 3/7/7). Diagnostics on admission to our unit were similar compared to twin I (see Figure 1b).

Explorative laparotomy on *day 11* revealed complete volvulus with partial gangrene of the ileum. Therefore, 6 cm of ileum were resected and a T drainage was placed in order to decompress the small intestine. During all of the surgical procedures he exhibited circulatory and renal failure requiring high dose vasopressors and diuretics.

**Figure 3:**
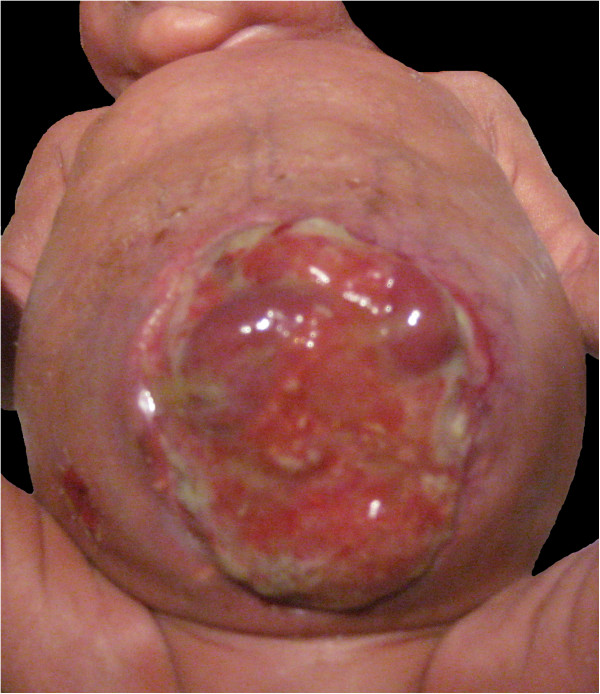
Abdomen of twin II after repeated unsuccessful surgery.

After failing to pass any meconium further surgery with adhesiolysis and transformation of T drainage to ileostoma was performed on *day 25*, which showed stenosis of colon ascendens and partial infarction of the small intestine. Due to distension the abdomen had to be left open (see Figure 3). Using prokinetic drugs (erythromycine, neostigmine) and enemas with N-acetylcysteine some passing of stool could be achieved.

When finally off the ventilator on *day 37*, twin II developed two spontaneous perforations next to the duodenum, which led to fistulas in the surface of the laparostoma. Without further surgical options at this time he received permanent parenteral nutrition via central venous lines, which led to three septic episodes.

Moreover, after just *6 weeks of treatment* a severe hepatopathy with massive elevation of liver enzymes and bilirubin (max. 30 mg/dl, direct 19 mg/dl) became evident. At an age of *4 months* liver synthesis function significantly declined necessitating daily substitution of plasma and albumin. A liver biopsy during next surgery proved 50% fibrosis.

Finally, the last option was the reconstruction of the continuity of the intestine. Unfortunately, soon after successful adhesiolysis and anastomosis of the remaining bowel *4 months after birth* further spontaneous perforations occurred and we decided to stop therapy in compliance with the parents and after ethical review.

Cystic Fibrosis Transmembrane Conductance Regulator (CFTR) mutation analysis in both twins had been performed after the first surgery, initially using the Cystic Fibrosis v3Genotyping Assay (Abbott Laboratories, Wiesbaden, Germany) and subsequently by Sanger sequencing of the entire coding region (27 exons) and 50 bp of flanking intronic sequences of the gene. Mutation analysis showed compound heterozygosity for the common CFTR mutation p.Phe508del (F508del or ∆F508, c.1521_1523delCTT at the DNA level) and another, as yet unknown mutation, p.Cys524Tyr (C524Y, c.1571G > A at the DNA level), both in exon 11. Mutation analysis in the parents revealed that the father was heterozygous p.Phe508del mutation, and the mother was a heterozygous carrier of the c.1571G > A mutation, thus confirming that the mutations occurred on different alleles (in trans) in the patients. Both the PolyPhen2 (http://genetics.bwh.harvard.edu/pph2/) and Mutation Taster software (http://neurocore.charite.de/MutationTaster/) classified the c.1571G > A mutation as protein damaging.

## Discussion

The impact of compound heterozygosity on disease course seems to depend on exact genotypes, as the milder mutation seems to be dominant and lead to milder courses [5]. Compound heterozygote ∆F508 patients differ from homozygote patients in degrees of pancreas insufficiency and sweat chloride levels, but not in the incidence of MI or lung function [6,7]. Especially the pancreas status of CF patients has been related to certain genotypes [7].

Nevertheless, there have been described associations of mutations like 621 + 1G- > T or G551D with the incidence of MI [6,7]. In contrast, Zielinski et al. excluded specific MI related CF mutations, but reported a higher incidence of MI in patients with a CF modifier locus on chromosome 19q13 (CFM1 gene, encoding for a calcium-dependant chloride channel) [5,8]. Further modifier loci (e.g. beta-defensins) have been reported and seem to be equally important in penetrance of MI and in lung function as disease defining gene mutations [5,9].

We have found a novel *CFTR* mutation which has not been previously described (http://www.genet.sickkids.on.ca/cftr/) and also not been reported as a variant in the 1000 Genomes Database (http://browser.1000genomes.org/index.html). A different, nonsense, mutation affecting the same codon, c.1572C > A (p.Cys524*), has previously been described in a CF patient [10]. Considering the position of the mutation in the region encoding the first nucleotide binding domain of the protein, it is highly likely that this mutation, together with F508del, is responsible for the CF in our patients.

## Conclusions

In conclusion we report on two extremely preterm male twins who were compound heterozygous for a potentially pathogenic, novel CF mutation, which might explain the severity of their disease despite early treatment. Both suffered from sequelae of prematurity and early onset CF, which led to multi organ dysfunction. Preterm infants with MI have to be evaluated for CF. Neonatal screening for CF might contribute to early diagnosis and treatment. Disease modifying loci have to be considered next to genotypes when defining severity and comorbidities of CF.

### Consent

Written informed consent was obtained from the parents for publication of this Case report and any accompanying images. A copy of the written consent is available for review by the Editor of this journal.

## Abbreviations

CF: Cystic fibrosis; MI: Meconium ileus; CFTR: Cystic fibrosis transmembrane conductance regulator; CFM1: Cystic fibrosis modifier 1.

## Competing interests

The authors declare that they have no competing interests.

## Authors’ contributions

AP conceived of and drafted the manuscript and was attending physician. DMR carried out and interpreted the genetic analysis and reviewed the manuscript. KDR was attending surgeon and reviewed the manuscript. CO carried out and interpreted the histologic studies. PG reviewed the manuscript. US carried out and interpreted the radiologic studies. RH was attending physician and helped to draft and review the manuscript. All authors read and approved the final manuscript.

## Pre-publication history

The pre-publication history for this paper can be accessed here:

http://www.biomedcentral.com/1471-2431/14/13/prepub
